# Supplementary figures and images for: Low immunogenicity of common cancer hot spot mutations resulting in false immunogenic selection signals
Source: PLoS Genet. 2021 Feb 8;17(2):e1009368. doi: 10.1371/journal.pgen.1009368 (PMC7895404; doi:10.1371/journal.pgen.1009368)

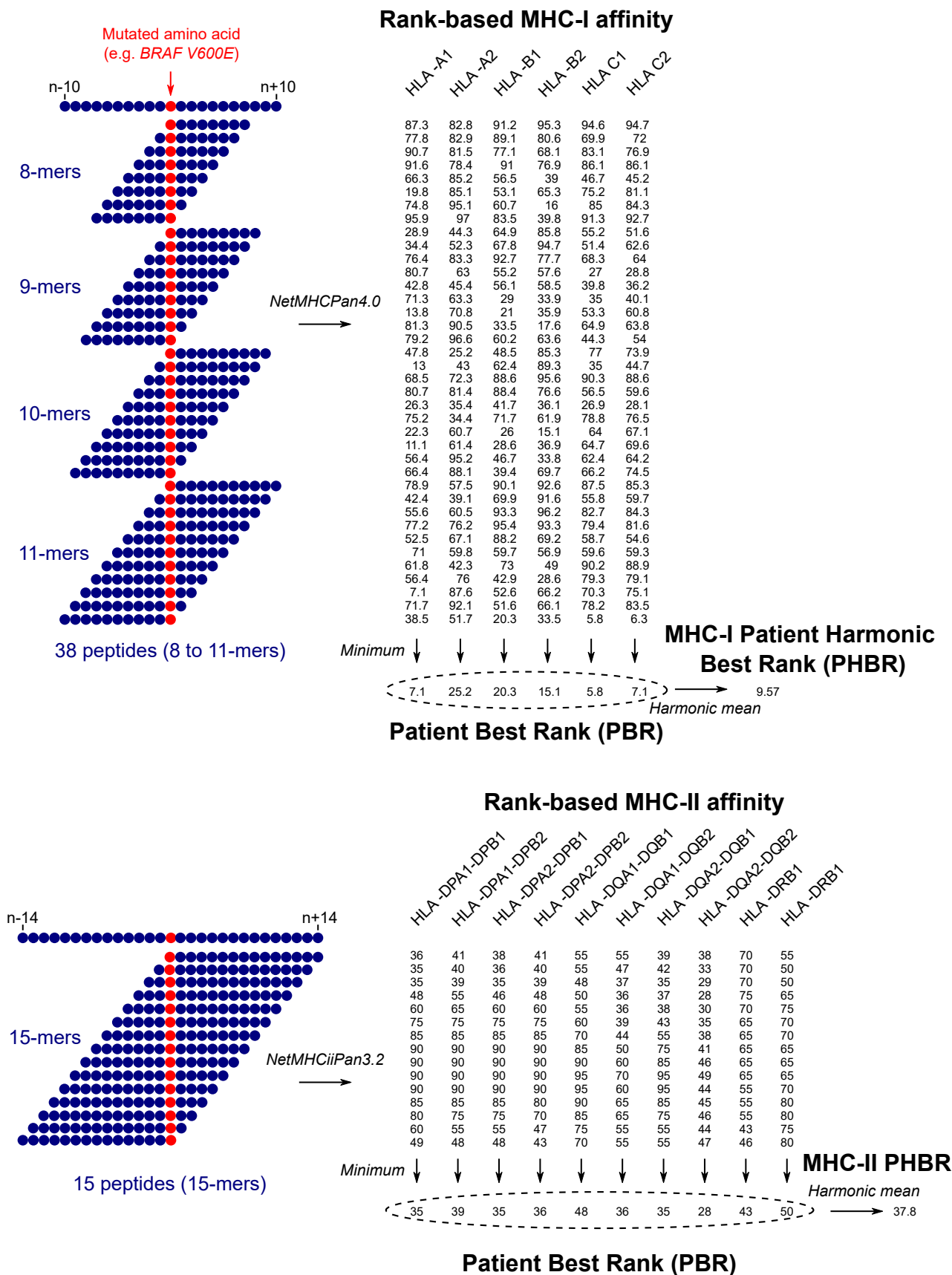

Suppl. figure 1

Supplement: S1 Fig — Perimutational amino acid sequences (21-mers for MHC-I, 29-mers for MHC-II) were derived using EnsemblDb. Based on these sequences, all 8 to 11-mers (MHC-I, top) and 15-mers (MHC-II, bottom) containing the mutation were determined as illustrated. For each peptide and MHC-I/MHC-II allele, the HLA affinities were predicted and quantified using a rank-based score. The Patient Best Rank (PBR) was then obtained from the best binding (lowest) ranks. Finally, the Patient Harmonic Best Rank (PHBR) was calculated from the PBRs. Numbers were derived from BRAF V600E (prototypical MHC genotypes, see Methods) and given for illustration purposes. Note that MHC-II DP and DQ genes form heterodimers between alpha and beta subunits. (PDF) [file pgen.1009368.s001.pdf]

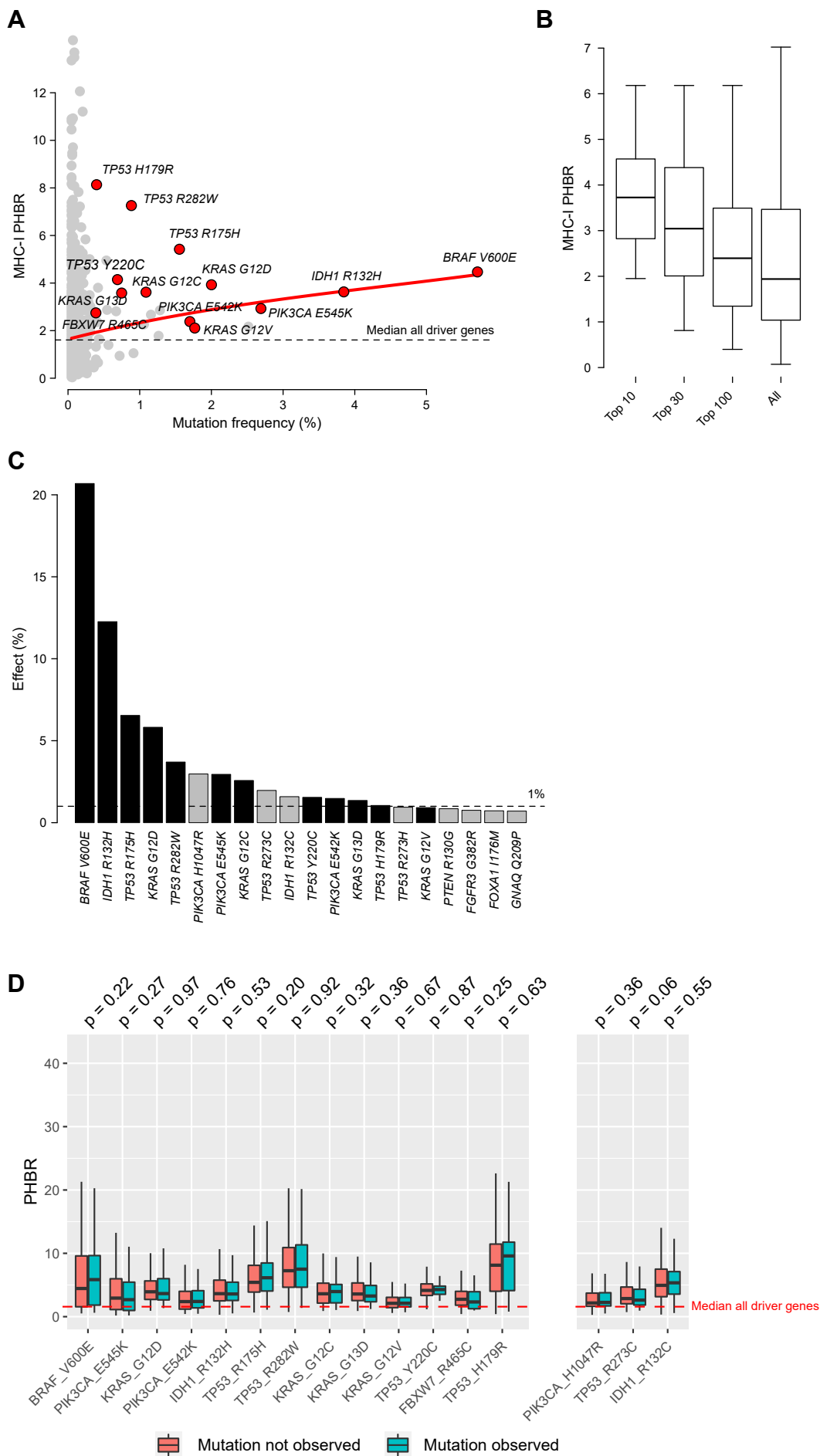

Suppl. figure 2

Supplement: S2 Fig — PHBR scores were calculated considering TCGA MHC-I genotypes (as in Fig 1). (A) Scatter plot showing the correlation between mutation frequency and median PHBR values. Thirteen weak HLA affinity mutations, as identified in Fig 3, are highlighted in red and labelled. Loess regression line shown in red. Median PHBR value from all 688 analyzed driver genes indicated by dashed line. (B) Box plot comparing PHBR values for the 10/30/100 most frequent mutations as indicated. (C) Bar plot showing the effect size (%) of the 20 driver mutations in the dataset with the largest effect. See Fig 3 for details on effect size calculations. Bars corresponding to the 13 weak HLA affinity mutations from the main analysis are colored in black. (D) Comparison of PHBR scores between patients with observed and unobserved mutations. Box plots show scores for the 13 weak HLA affinity mutations identified in the main analysis (left) and the 3 additional mutations with minimal effect size of 1% as identified in panel C (right). Box plots indicate median values and lower/upper quartiles with whiskers extending to 1.5 times the interquartile range. P values calculated using Wilcoxon rank-sum test. (PDF) [file pgen.1009368.s002.pdf]

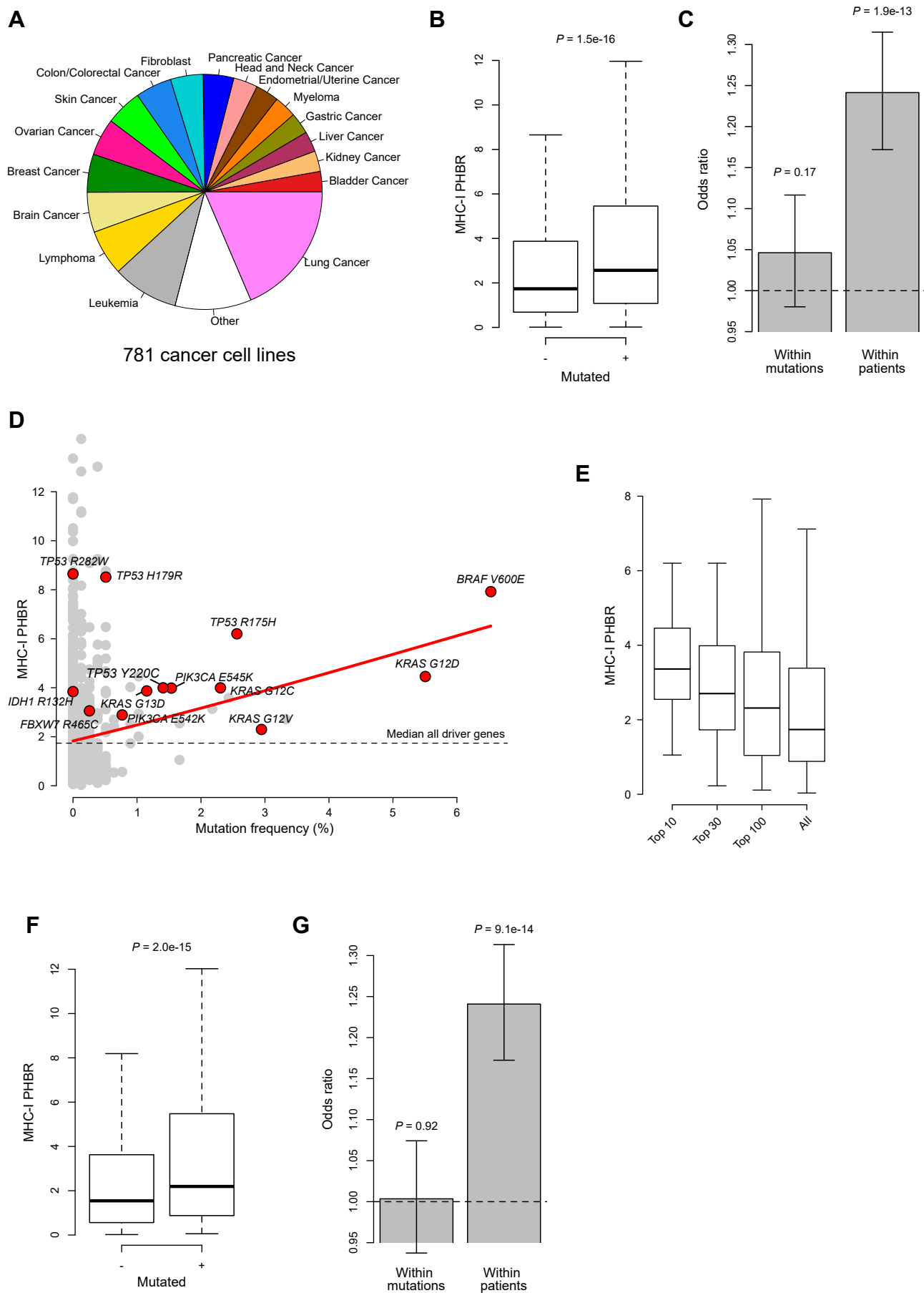

Suppl. figure 3

Supplement: S3 Fig — PHBR scores were calculated for all hot spot driver mutations in cancer cells from the Cancer Cell Line Encyclopedia (CCLE), for which HLA allele calls were available. Analysis in (A-E) are based on sample-specific HLA alleles, while (F-G) are based on the prototypical MHC-I genotype. (A) Pie chart showing the different primary tumors where the cell lines were derived from. (B) Box plots compare PHBR values between observed (+) and unobserved (-) mutations. (C) Logistic regression analysis between log PHBR (observed variable) and mutation status (response variable). Analysis was performed using both the within-patient and within-mutation regression model as indicated (see main analysis for details). (D) Scatter plot showing correlation between mutation frequency and median PHBR values. Thirteen weak HLA affinity mutations, as identified in Fig 3, are highlighted in red and labelled. Loess regression line shown in red. Median PHBR value from all 688 analyzed driver genes indicated by dashed line. (E) Box plot comparing PHBR values for 10/30/100 most frequent mutations as indicated. (F-G) Similar as panels (B) and (C) but with PHBR values calculated based on the prototypical MHC-I genotypes. Box plots indicate median values and lower/upper quartiles with whiskers extending to 1.5 times the interquartile range. P values calculated using Wilcoxon rank-sum test. (PDF) [file pgen.1009368.s003.pdf]

**A**

### Most frequent HLA alleles

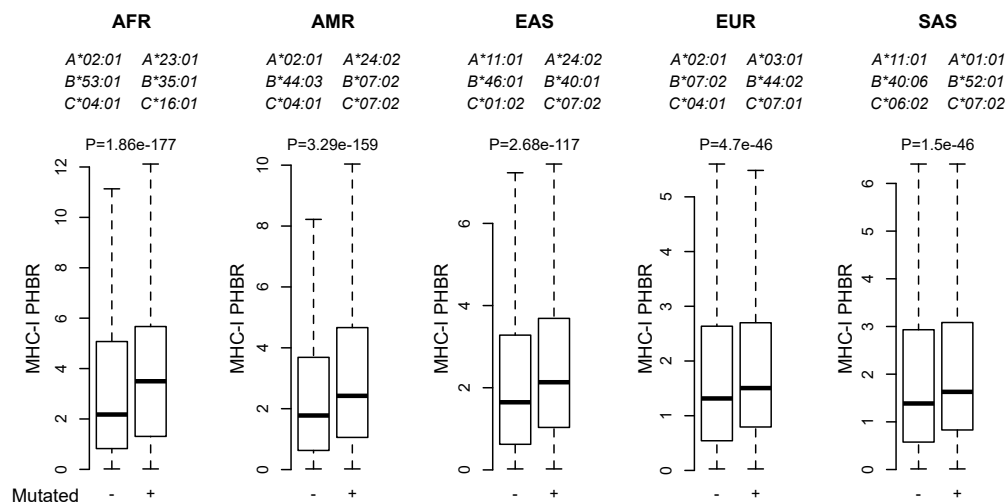

**B**

### Rare (unobserved) HLA alleles

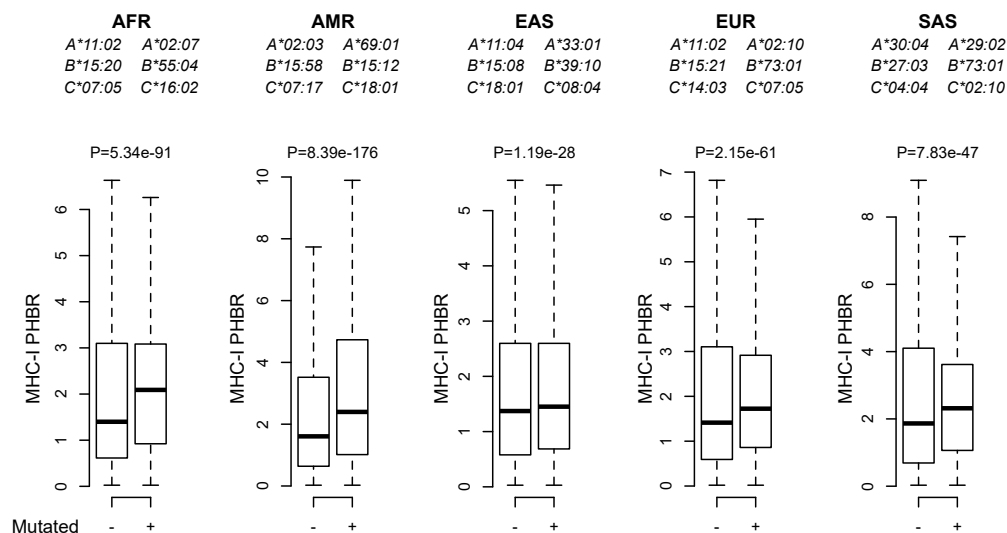

Supplement: S4 Fig — PHBR scores were calculated considering different MHC-genotypes as indicated. Box plots compare PHBR scores between observed (+) and unobserved (-) mutations. Mutations were derived from the virtual patient mutation matrix used in the main analysis. (A) MHC-I genotype composed of the 2 most frequent HLA-A, HLA-B and HLA-C alleles for different populations from the 1000 genomes project. (B) MHC-I genotype composed of 2 random HLA-A, HLA-B and HLA-C alleles that were never observed in the indicated population. Box plots show median values and lower/upper quartiles with whiskers extending to 1.5 times the interquartile range. P values calculated using Wilcoxon rank-sum test. AFR, African; AMR, Ad Mixed American; EAS, East Asian; EUR, European; SAS, South Asian. (PDF) [file pgen.1009368.s004.pdf]

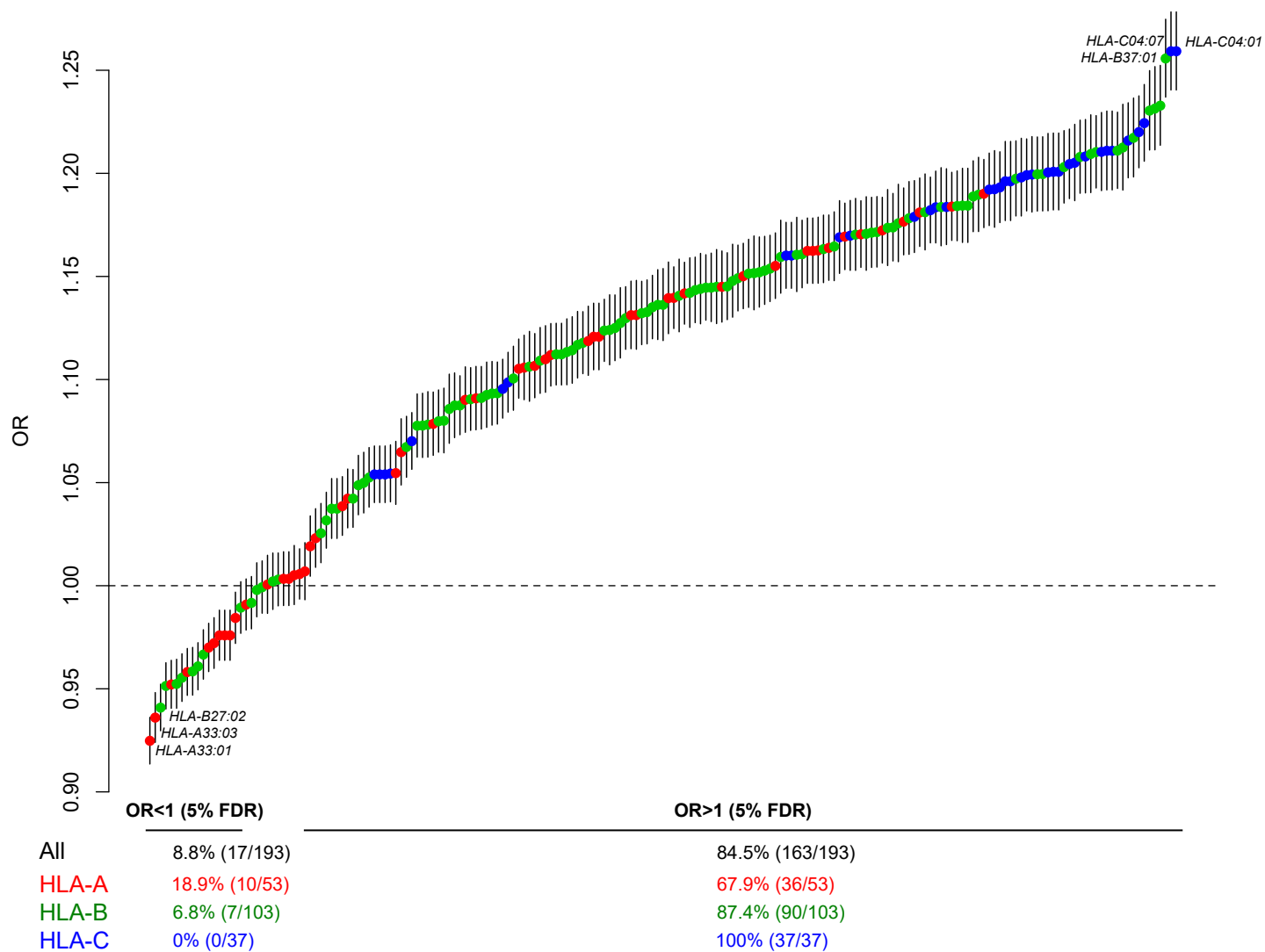

Suppl. figure 5

Supplement: S5 Fig — Patient Best Rank (PBR) scores were calculated for each of 193 different alleles that were called minimally once in the TCGA dataset. The within-patient logistic regression analysis was performed considering the mutation matrix used for the virtual patient main analysis. Plot shows odds ratios (OR) with 95% confidence intervals for each allele with HLA-A, HLA-B and HLA-C indicated by colors and alleles with highest/lowest OR labelled. Alleles with OR above/below 1 (determined at 5% FDR) with corresponding frequencies are indicated below the plot. (PDF) [file pgen.1009368.s005.pdf]

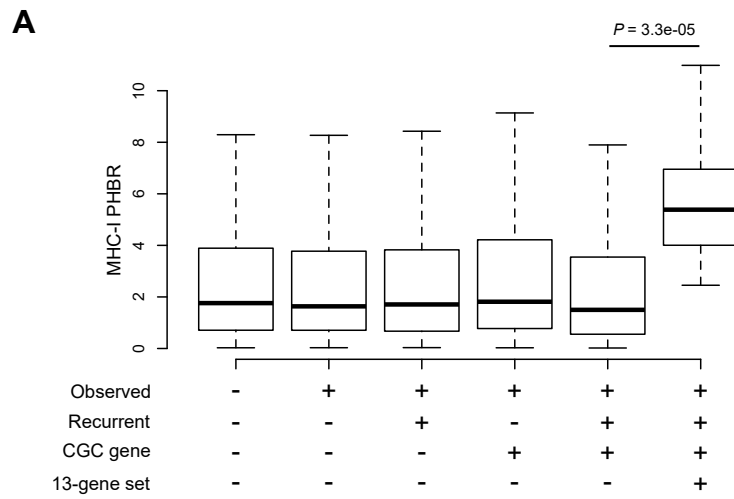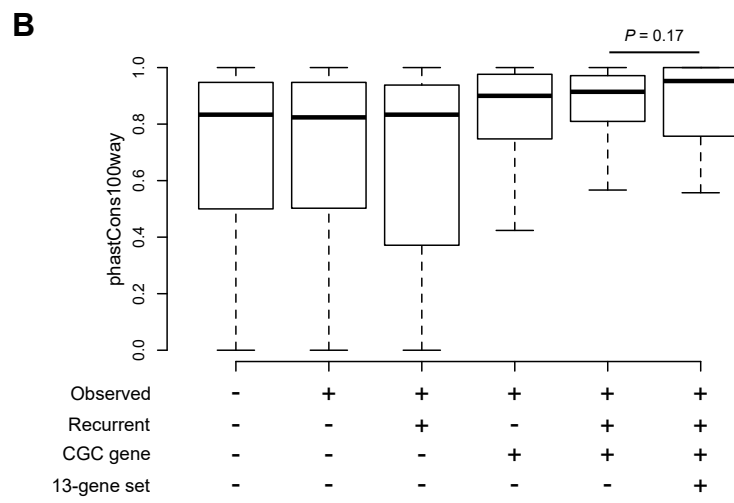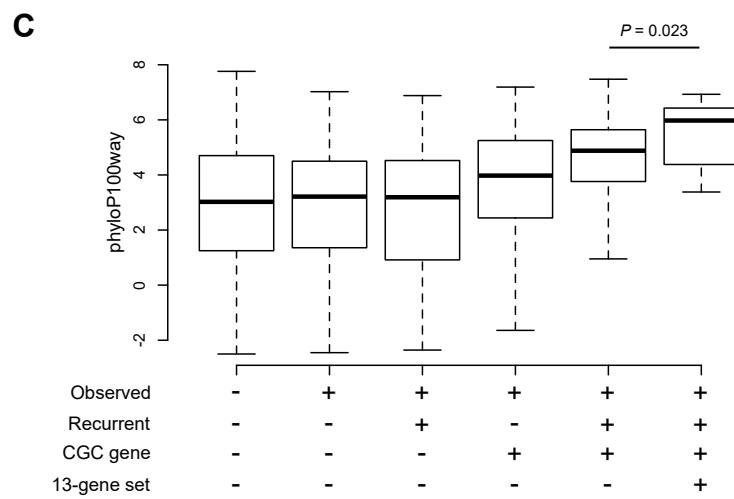

Suppl. figure 6

Supplement: S6 Fig — Box plots compare MHC-I PHBR scores (A) and 2 evolutionary conservation scores (B, pastCons; C, phyloP) between different somatic mutation datasets. From left to right and as indicated: 500 random missense mutations that were never observed in the TCGA dataset; 500 random missense mutations that were not recurrent (occurred once in the TCGA dataset) and are not known as a Cancer Gene Census (CGC) gene; 500 recurrent missense mutations that are not known as CGC genes; 500 non-recurrent missense mutations in CGC genes; 688 recurrent missense mutations in CGC genes (the dataset used for the main analysis) that are further divided depending on the presence of the gene in the 13-gene weak HLA affinity mutation dataset identified in the main analysis. phastCons (B) and phyloP (C) were derived from multiple alignments between 100 vertebrate species and calculated for the genomic region extending +/- 10 base pairs from the mutation (21 base pairs in total). Box plots indicate median values and lower/upper quartiles with whiskers extending to 1.5 times the interquartile range. P values calculated using Wilcoxon rank-sum test. (PDF) [file pgen.1009368.s006.pdf]
