# Supplementary material for: Low immunogenicity of common cancer hot spot mutations resulting in false immunogenic selection signals
Source: PLoS Genet. 2021 Feb 8;17(2):e1009368. doi: 10.1371/journal.pgen.1009368 (PMC7895404; doi:10.1371/journal.pgen.1009368)
Supplement: Attachment — Submitted filename: PGENETICS_D_20_01663_responseToReferees.pdf [file pgen.1009368.s007.pdf]

Point-by-point response

Manuscript PGENETICS-D-20-01663

"Low immunogenicity of common cancer hot spot mutations resulting in false immunogenic selection signals"

Dear reviewers, dear editor,

Below you can find our point-by-point response to the reviewers' comments on our manuscript entitled "*Low immunogenicity of common cancer hot spot mutations resulting in false immunogenic selection signals*".

We are most grateful to the reviewers for providing appropriate and constructive comments to our manuscript and we sincerely thank everyone for their time and effort. The points have been addressed to the best of our abilities, and we believe that this has led to the study being considerably improved.

<u>Main changes/additions:</u>

- We have extended our core analysis to the real patient TCGA data (suppl. Fig. 2) and also added a new analysis on cancer cell line data (suppl. Fig. 3).
- We confirmed our results using both common (prototypical) and rare genotypes from 5 different populations (suppl. Fig. 4).
- An evolutionary conservation analysis was added (suppl. Fig. 5).

Note that we have listed Tom Luijts as additional co-author. He was involved in several analyses that were performed during revision, more specifically the analysis of the cancer cell line dataset and the summarizing figure 6.

For clarity, reviewer's remarks are shown below in black while our responses are in blue.

Reviewer #1

This article is very interesting, well written and clearly argues for a critical re-evaluation and interpretation of predictions based on HLA-binding affinity when studying immunoediting. Like all papers that challenge a dogma, this paper could stir some controversy. Nonetheless, I found that the analyses were solid and yielded several nuggets of information on common driver mutations. In my opinion, this article should be of great importance to the fields of cancer genetics and cancer immunology.

We thank the reviewer for these appreciating comments.

Minor Point:
Line 59-60: What are the three regions that correspond to the six mentioned loci?

We are referring to the main genes that determine the MHC-I and MHC-II genotype respectively, 3 for each, six in total. To make avoid confusion we rephrased as following:

> *The MHC-I and MHC-II genotype is each determined by 3 highly polymorphic HLA gene regions on chromosome 6 (MHC-I: HLA-A, -B and -C; MHC-II: HLA-DP, -DQ and -DR), implying thousands of possible HLA genotypes in a population [13].*

Reviewer #2

The paper by Claeys, Marchal and Van den Eynden addresses the tendency of known cancer driver mutations to not be very well presented by the MHC complex. The authors challenge the common argument that this is due to selection bias by the immune system. They first show that observing a specific driver mutation is unrelated to the specific HLA genotype of the individual, and indeed a few common mutations have low presentation leading to this effect. They then highlight some of these specific mutations and show why they decrease the peptides' HLA affinity generally.

The paper is based on sound and thorough statistical analysis. The results address a current research question in a timely manner. The paper is convincing and interesting to researchers interested in the interaction of the immune system and cancer.

We thank the reviewer for these positive comments.

My main comment is that the analysis in some places can be obfuscating and unintuitive, especially to non-statisticians. Some choices and assumptions are not explained clearly enough. I suggest making some of the claims and arguments more transparent.

We agree some parts might have been hard to follow for non-statisticians. We made several efforts to increase the readability of the manuscript, mainly on the logistic regression analysis, which is now introduced as following in the results section:

> *As an alternative approach to quantify the PHBR difference between observed and unobserved mutations, we determined the association between mutation probability and PHBR scores using a logistic regression approach as suggested previously [22]. Two different models were used: 1) to determine how mutation probability correlates to PHBR scores within patients (rows of the mutation matrix), a within-patient model was used that controlled for mutational burden variation between patients; 2) to determine how mutation probability correlated to PHBR scores across patients (columns of the mutation matrix), a within-mutation model was used that controlled for mutation frequency variation between mutations. In line with the randomization approach, a positive correlation was found between mutations and PHBR scores using the within-patient model (odds ratio (OR) = 1.19, P = 1.66e-87), but not when using the within-mutation model (OR = 1.01, P = 0.21; Fig 1D). Again, these findings are not in agreement with selection processes, which are expected to lead to comparable results when using both models.*

Specific points:

1) The main result was the association between the frequency of driver mutations and the PHBR score. It would be both interesting and helpful to show a scatter plot of these two quantities for the TCGA data, maybe on a log scale, highlighting the (13) mutations most contributing to the association. This would support the statistical analysis already done in a clear manner.

We agree that highlighting these 13 genes on fig. 2D is helpful and changed the figure as suggested.

We have also added a similar scatter plot for real patients (considering the actual HLA genotypes from TCGA) in supplementary figure 2A. Results were very similar to the analysis with the prototypical genotype.

2) Most of the results were based on HLA binding analysis using a prototypical HLA genotype. While the authors did show that the low presentability of the mutations doesn't seem to be HLA specific, it's not clear if certain rarer choices of HLA alleles wouldn't change this. Specifically I would be interested to see if the results for the weaker HLA affinity for the 13 driver mutations hold for different choices of HLA, maybe a random pick or based on different populations.

We have extended our analysis and showed similar results when using the most common HLA alleles from 5 different populations (Suppl. Fig. 4A). Also changing to rare HLA alleles from these populations didn't alter our conclusions (Suppl. Fig. 4A). Relatedly, we now also demonstrate that the HLA affinity differences we describe are a general property of the majority of HLA alleles (Suppl. Fig. 5). We describe this in the results section as following:

> *We next determined whether the observed PHBR differences were attributable to the specific composition of the prototypical MHC genotype or rather represent a more general HLA allele property. When the underlying MHC-I genotype was altered to the 2 most frequent HLA-A, HLA-B and HLA-C alleles from 5 other populations (European, American, South-Asian, East-Asian or African), PHBR values were always higher for observed than unobserved mutations (S4 Fig. A). Also considering HLA alleles with rare population frequencies did not change our conclusions (S4 Fig. B), suggesting that the PHBR difference is related to a general property of the individual HLA alleles. Indeed, an allele-based logistic regression analysis (within patient model) on 193 different alleles confirmed that the ORs were significantly above 1 (5% FDR) for the majority (163/193, 84.5%) of all analyzed alleles. Notably, differences were observed between the HLA genes, with 100% of HLA-C alleles, 87.4% of HLA-B alleles and 67.9% of HLA-A alleles having ORs above 1 (S5 Fig.).*

3) The 688 mutations used for the binary matrix were recurrent missense mutations, so all of the them were observed in at least some of the patients. Can the authors say anything about mutations which were really not observed, by simulating them and checking HLA affinity?

In suppl. Fig. 5, we have added an analysis on different sets of mutations, including 500 random unobserved mutations, as suggested by the reviewer. HLA affinities were similar to other sets of mutations and only the group of 13 identified mutations showed weaker affinities.

4) Line 169: why only 150 possible amino acid substitutions in the human?

In theory there are 400 (20x20) possible amino acids substitutions. However, due to redundancy of the genetic code and the specific codon compositions, only 150 occur in the human genome. This number was derived from a simulation of all possible point mutations in the genome. We rephrased this part of the methodology to clarify:

> *All point mutations in the human genome were simulated, resulting in 150 possible missense substitutions. We randomly sampled 100 substitutions from each substitution type and generated a dataset containing information for 15,000 substitutions in total.*

5) Line 172: Do Glu and Gly suppose to be the same amino acid?

This is indeed a typo (should be Glu, we corrected this). Thanks for noting this.

6) Line 191: is the d in “dPHBR” should be a delta sign?

Indeed, we corrected this.

7) Line 192: it is not clear how the substitution effect is neutralized by focusing on PHBR values from wild type peptides.

We agree this is a confusing sentence. We were referring to the fact that the higher PHBR values are not solely caused by the substitution itself (as measured by ∆PHBR), but also by the amino acid sequence context of the wild type peptide. We rephrased as following:

> *Furthermore, when focusing on PHBR values from wild type peptides only, the PHBR score difference between observed (median PHBR 1.91) and unobserved (median PHBR 1.51) mutations is still present (P = 1.1e-34; Fig 5A), …*

8) Line 202: what are the 38 PHBR-determining peptides and how are they determined?

We were referring to the 38 different peptides that can contain the substitution in 8-11 mers, as explained in the methodology and Fig. S1. We added reference to clarify for the reader:

> *As a first, general approach we checked for an association between the percentage of each amino acid in all 38 PHBR-determining peptides (see Methods and Fig. S1) and the mutation frequency.*

9) Line 347: “This data” should be “These data” (data is plural)

We corrected this.

Reviewer #3

In this paper Claeys et al. challenge findings from two previous high-impact studies that suggest that immune pressure may be modulating the evolution landscape of cancers. As a follow up to their previous work recently published in Nature Genetics, the authors here focus on a set of key annotated driver mutations. They first show that indeed in line with previous reports, there does indeed seem to be an HLA related signal in these mutations, which suggests that they may driven by immune pressure. To challenge these findings, the authors conduct a set of simulation experiments in which they:

1. Generate a random set of virtual patients (n=10,295) all with a single HLA genotype (key to their argument) and randomly sampled cancer genomes using a site independent sampling scheme for each of the 688 driver mutations based on their individual frequencies.

2. Show that even in this genotype independent set they can find differences between the binding scores (PHBRs) of observed and unobserved mutations.

3. Use a leave-one-out analysis to identify specific key mutations that likely explain away this effect- identifying 13 such mutations

3. Dig into some of these mutations in detail attempting to provide alternate explanations as to why such mutations may be caused unrelated to HLA related pressure. Most interestingly here, they show that mutations of different AA have different effects on the binding scores, and that some tend to increase (or decrease) HLA binding when mutated.

Overall this is a very interesting paper that is well written that challenges important findings based on HLA binding predictions that suggest that cancer evolution may be influenced by immune mediated pressure. The data presented here, indeed provides significant evidence for alternate possible explanations of these findings, which requires revisiting some of these studies. As such, it is clearly an important paper.

I particularly liked the fact that the authors do not argue about the quality of the HLA binding predictors, but instead show alternate possible explanations to these findings, in line with their previous work.

We thank the reviewer for this accurate summary and the positive comments on our study.

However, seeing as this is a second paper in this line of work, I would urge the authors to dig a little deeper into their analysis to possible strengthen their claims:

Major comments:

1. The authors once more use a prototypical HLA genotype which the most common HLA class I and class II alleles. Are the effects described here also true for other less common HLA

genotypes? i.e. if the authors were to select other (random?) genotypes would similar results be obtained? I am asking this since as the authors note, immmunotherapy does actually work very well in some patients, for reasons that are poorly understood, underlying the potential importance of T-cell mediated immune pressure on tumors.

The effects we describe seem to be a general property of most HLA alleles. To illustrate this, we have extended our analysis and show similar results when using the most common HLA alleles from 5 different populations (Suppl. Fig. 4A). Also changing to rare HLA alleles from these populations didn't change our conclusions (Suppl. Fig. 4B). Relatedly, we now also demonstrate that the HLA affinity differences we describe are a general property of the majority of HLA alleles (Suppl. Fig. 5). We describe this in the results section as following:

> *We next determined whether the observed PHBR differences were attributable to the specific composition of the prototypical MHC genotype or rather represent a more general HLA allele property. When the underlying MHC-I genotype was altered to the 2 most frequent HLA-A, HLA-B and HLA-C alleles from 5 other populations (European, American, South-Asian, East-Asian or African), PHBR values were always higher for observed than unobserved mutations (S4 Fig. A). Also considering HLA alleles with rare population frequencies did not change our conclusions (S4 Fig. B), suggesting that the PHBR difference is related to a general property of the individual HLA alleles. Indeed, an allele-based logistic regression analysis (within patient model) on 193 different alleles confirmed that the ORs were significantly above 1 (5% FDR) for the majority (163/193, 84.5%) of all analyzed alleles. Notably, differences were observed between the HLA genes, with 100% of HLA-C alleles, 87.4% of HLA-B alleles and 67.9% of HLA-A alleles having ORs above 1 (S5 Fig.).*

2. Can the authors show similar effects using a dataset of non-virtual patients? i.e. can they simply pickup a random HLA geneotyped cohort and use the actual patients in that cohort to replicate their findings - this in my mind would be a stronger statement than the one using a single HLA genome - simply because there are likely evolutionary related effects that have selected for these frequent HLA alleles.

The analysis as shown in Fig. 1 was solely based on real patient TCGA data (considering patient-specific HLA alleles). We have now extended this analysis and show the same correlations with mutation frequency (Suppl. Fig. 2A-B) and similar results using the leave-one-out analysis (Suppl. Fig. 2C) as with the prototypical MHC genotypes. To demonstrate that any mutation-specific difference was not caused by selection, we compared the patient-specific PHBR scores from patients with and without each identified mutation (Suppl. Fig. 2D). This is described in the results section as following:

> *Similar results were obtained when PHBR scores were calculated considering the TCGA patient-specific MHC-I genotypes (S2 Fig.).*
>
> *…*

Repeating the leave-one-out analysis on the TCGA dataset directly (considering each patient-specific MHC-I genotype) confirmed the contribution of 11 of the 13 identified mutations to the PHBR difference, with another one just below the detection threshold of 1% (KRAS G12V, 0.92%; S2 Fig. C). PHBR scores were always similar between patients with observed and unobserved driver mutations, confirming the absence of any detectable selection pressure on the genotype level (S2 Fig D).

Additionally, we added a new analysis using genomic data from the Cancer Cell Line Encyclopedia, considering the actual genotypes, showing highly similar results (Suppl. Fig. 3). This is described in results section as following:

An orthogonal analysis using genomic data from a diverse set of 781 cancer cell lines yielded analogous results: higher PHBR scores for the most frequent mutations (median PHBR 3.36 for the top 10 versus 1.73 for the complete dataset), increased odds ratio using the within-patient model (1.24, P=1.9e-13) and an effect that was also observed when using the prototypical MHC genotype (S3 Fig.).

3. The authors should also analyze the evolutionary conservation of the key driver mutations, as an alternate explanation to the association with HLA binding. Previous work has shown that HLA tend to bind to evolutionarily conserved regions of human and viral proteins, and if the key driver mutations are indeed in highly conserved sites, they may be under immune monitoring simply as a side-effect of the optimization of the immune system to focus on these regions in pathogens and self.

We thank the reviewer for this suggestion and now analyzed evolutionary conservation using both phastCons and phyloP scores in Suppl. Fig. 6B-C. There was some interesting trend for better conservation in the genomic regions containing the 13 mutations, as compared to the regions containing the other mutations (significant for phyloP, not for phastCons), although we believe it is hard to conclude anything from this observation at this stage. We added a section in discussion related to this observation:

We showed that the weaker HLA affinities in peptides translated from these 13 driver mutations were a general HLA allele property. It remains an open question whether these altered affinities are a simple epiphenomenon, resulting from the more efficient oncogenic mechanisms, or, alternatively, are related to differing germline evolutionary pressures (e.g. related to host-pathogen coevolution [33]). While a trend was observed towards better evolutionary conservation for the genomic regions containing the 13 mutations, any putative causal relationship remains to be determined.
